# T115. REASONING BIAS, WORKING MEMORY PERFORMANCE, AND A TRANSDIAGNOSTIC PHENOTYPE OF AFFECTIVE DISTURBANCES AND PSYCHOTIC EXPERIENCES IN THE GENERAL POPULATION

**DOI:** 10.1093/schbul/sby016.391

**Published:** 2018-04-01

**Authors:** Christian Rauschenberg, Ulrich Reininghaus, Margreet ten Have, Ron de Graaf, Saskia van Dorsselaer, Nicole Gunther, Lotta-Katrin Pries, Sinan Guloksuz, Rajiv Radhakrishnan, Maarten Bak, Jim van Os

**Affiliations:** 1 Maastricht University; 2 Trimbos Institute; 3 Yale University School of Medicine; 4 University Medical Center Utrecht

## Abstract

**Background:**

There is robust evidence that reasoning biases such as a tendency of jumping to conclusions (JTC) as well as cognitive deficits are associated with psychosis, but evidence on their association with affective disturbances remains inconclusive. Recent findings also suggest a transdiagnostic phenotype of co-occurring affective disturbances and psychotic experiences. This study aimed to investigate whether JTC bias and decreased working memory performance are associated with the co-occurrence of affective disturbances, psychotic experiences (PEs), and psychosis-related help-seeking behaviour (HS) in the general population.

**Methods:**

The second Netherlands Mental Health Survey and Incidence Study (NEMESIS-2). Trained interviewers administered the Composite International Diagnostic Interview (CIDI) at three time points in a representative general population sample, with N=4.596 individuals who completed all assessments. The beads task and digit-span task were completed to assess JTC bias and working memory performance, respectively. CIDI was used to measure affective disturbances (i.e. depression, anxiety, mania) and an add-on instrument to measure PEs and HS.

**Results:**

We found that, compared to individuals with neither affective disturbances nor PEs, JTC bias was more likely to be present in individuals with co-occurring affective disturbances, PEs, and HS (moderate psychosis [1–2 PEs]: relative risk ratio [RRR]=1.23, 95% CI 1.03 – 1.48, p=0.023; high psychosis [3 or more PEs or HS]: RRR=1.66, 95% CI 1.26 – 2.19, p<0.001) in models adjusted for socio-demographic characteristics and socio-environmental factors. However, when we additionally adjusted for working memory performance this association was attenuated (moderate psychosis: RRR=1.17, 95% CI 0.98 – 1.41, p=0.088; high psychosis: RRR=1.57, 95% CI 1.19 – 2.08, p=0.002). In line with previous findings, there was no evidence that JTC bias was more likely to occur in individuals with sole presence of affective disturbances (RRR=1.03, 95% CI 0.94–1.13, p=0.492). Further, there was some evidence of a dose-response relationship, as JTC bias was progressively more likely to occur in individuals with affective disturbances as the level of PEs increased or HS was reported (high vs. moderate psychosis: p=0.052). In contrast, compared to individuals with neither affective disturbances nor PEs, a decreased working memory performance was evident in all groups (i.e., affective disturbances only: RRR=0.94, 95% CI 0.90–0.98, p=0.006; PEs only: RRR=0.79, 95% CI 0.69–0.91, p=0.001; co-occurring affective disturbances and moderate psychosis: RRR=0.83, 95% CI 0.75–0.91, p<0.001; co-occurring affective disturbances and high psychosis: RRR=0.76, 95% CI 0.65–0.89, p=0.001).

**Discussion:**

Our findings suggest that JTC bias and decreased working memory performance may contribute to a transdiagnostic phenotype of co-occurring affective disturbances and PEs. Further, findings support the notion that JTC bias may be specifically associated with psychosis, including in those presenting a transdiagnostic phenotype, while a lowered working memory performance may represent a more broadly distributed vulnerability factor across various symptom domains. Overall, results point to the need to further investigate whether established mechanism and risk factors, described to be involved in the development and maintenance of psychosis, extend to transdiagnostic phenotypes to further corroborate proposed aetiological models and overcome shortcomings of focussing only on specific domains of mental health.

